# Direct-to-Patient Mobile Teledermoscopy: Prospective Observational Study

**DOI:** 10.2196/52400

**Published:** 2024-02-12

**Authors:** Winnie Fan, Gunnar Mattson, Amanda Twigg

**Affiliations:** 1 Department of Dermatology School of Medicine University of California San Francisco San Francisco, CA United States; 2 Department of Dermatology San Francisco Veterans Affairs Health Care System San Francisco, CA United States

**Keywords:** mobile teledermoscopy, teledermatology, direct-to-patient, full body skin exam, diagnostic concordance, mobile health, mHealth, dermoscopy, dermatology, dermatological, imaging, image, images, smartphone, lesion, lesions, skin, diagnostic, diagnosis, diagnoses, telehealth, telemedicine, eHealth

## Abstract

Direct-to-patient mobile teledermoscopy is a feasible and useful adjunct to smartphone imaging for monitoring patient-identified lesions of concern, achieving comparable diagnostic and management accuracy as in-office dermatology.

## Introduction

Teledermoscopy is promising for improving the diagnostic accuracy of store-and-forward consultations [1]. However, few studies have explored using direct-to-patient mobile teledermoscopy to bypass in-person imaging [2-4]. Within the Veterans Health Administration system, teledermatology involves in-person visits with trained imaging technicians. Dermoscopy is not universally used. This prospective observational study evaluates a direct-to-patient mobile teledermoscopy program at the San Francisco Veterans Affairs Medical Center (SFVAMC) on its effectiveness in diagnosing and managing patient-identified lesions of concern.

## Methods

### Recruitment and Implementation

**Figure 1:**
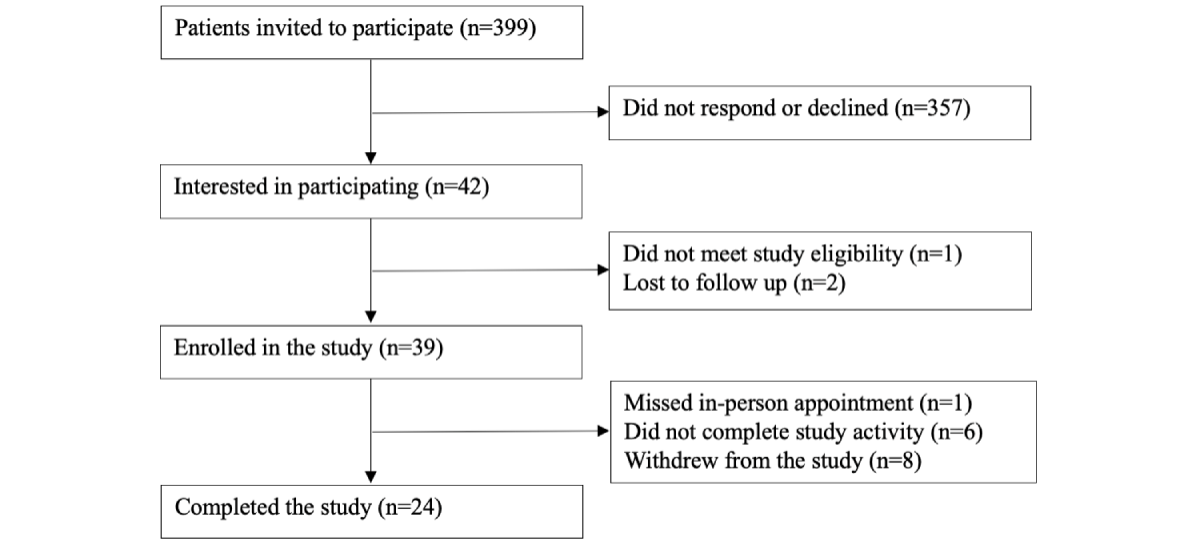
Study participant recruitment flow diagram.

Adults scheduled for full-body skin exams between May and August 2022 were recruited (Figure 1) and given a Sklip mobile dermatoscope, valued at US $99.99. They were instructed to image 1-3 lesions of concern using both smartphones and dermatoscopes. A teledermatologist reviewed all images for diagnosis, management, quality, and clinical utility. Clinical utility was defined as images that increased the teledermatologist’s confidence in diagnosis and management. A dermatologist different from the teledermatologist evaluated the same lesions in-office.

### Statistical Analysis

The degree of agreement was assessed using the percentage of agreement and Cohen κ (95% CI). Cohen κ values were interpreted using the scale developed by Landis and Koch [5]. Excel (Microsoft Corporation) was used for data collection and analysis.

### Ethical Considerations

This study was approved by the institutional review board (IRB) of the UCSF Human Research Protection Program and SFVAMC Research and Development Committee, IRB study number 21-33538. Participants provided informed consent with the option to opt out of the study. Participants were not compensated, and their data was anonymized and stored in a password-protected file.

## Results

This study included 24 participants (male: n=20, 83%; mean age 65.3, SD 14.9 years). The average distance between their home zip codes and SFVAMC was 54.9 (SD 77.1) miles. A total of 12 (50%) participants had a history of skin cancer: 10 with basal cell carcinoma, 5 with squamous cell carcinoma, 4 with melanoma, and 1 with melanoma in situ.

A total of 56 lesions were imaged: 9 (17%) on the head, 1 (2%) on the neck, 8 (15%) on the posterior trunk, 16 (30%) on the anterior trunk, 15 (28%) on the arms, and 3 (9%) on the legs. The teledermatologist rated most dermoscopic images (n=37, 66%) as acceptable to good quality. There was substantial agreement between the teledermatologist and in-person dermatologist in diagnoses and management (Table 1; κ=0.65, SE 0.13, 95% CI 0.39-0.91 and κ=0.67, SE 0.11, 95% CI 0.47-0.88, respectively). Most discordant diagnoses had concordant management (n=3, 60%).

Over 85% (n=48) of lesions were diagnosed as benign neoplasms. Two participants had additional lesions suspected of malignancy identified by in-office dermatologists, one of which was biopsy-proven basal cell carcinoma. Teledermatologists considered 59% (n=33) of smartphone images to have clinical utility, while 66% (n=37) of dermoscopic images provided additional utility when used alongside smartphone images.

For 65% (n=15) of participants who responded to a questionnaire, nondermoscopy smartphone imaging was easy, whereas 52% (n=12) reported mobile teledermoscopy as easy. Most (n=18, 78%) were willing to perform mobile teledermoscopy again. Barriers to dermoscopy use included difficulty performing with nondominant hand (n=1, 4%) and requiring assistance (n=5, 22%). All dermatoscopes were returned undamaged.

**Table 1:** Distribution of diagnoses and management by the teledermatologist and in-office dermatologists.

		Teledermatologist (n=56), n (%)	In-office dermatologist (n=56), n (%)
**Diagnostic category**			
	Benign	48 (85.7)	48 (85.7)
	Premalignant	1 (1.8)	3 (5.4)
	Malignant	0 (0.0)	0 (0.0)
	Infectious	0 (0.0)	1 (1.8)
	Inflammatory	7 (12.5)	4 (7.1)
	Neoplasm of uncertain behavior	0 (0.0)	0 (0.0)
**Management**			
	Monitor	44 (78.6)	43 (76.8)
	Cryotherapy	1 (1.8)	3 (5.4)
	Biopsy or excision	4 (7.1)	2 (3.6)
	Antibiotic	1 (1.8)	2 (3.6)
	Steroid/anti-inflammatory	6 (10.7)	6 (10.7)

## Discussion

### Principal Findings

Substantial agreement was found between the teledermatologists and in-office dermatologists, consistent with previous studies [2,6]. However, the wide CIs indicate the need for further studies with larger sample sizes and implementation improvements, especially for identifying life-threatening malignancies. We recommend providing patients’ medical history to teledermatologists. In one discordant case, a history of vitiligo could have differentiated from postinflammatory hypopigmentation. A recent study developed a checklist for mobile teledermoscopy image quality [7], which could be shared with patients to improve image quality. Because the teledermatologist had a lower threshold for biopsies, a follow-up office visit should be pursued when a procedure is recommended.

Given the high proportion of benign neoplasms in our study, teledermoscopy implementation for patient-identified lesions could lead to an increased burden for telediagnosis services. To increase the malignancy detection, we recommend providing patient education on high-risk features, such as the ABCDEs (asymmetry, border, color, diameter, and evolving) of melanoma or the 7-point checklist, before imaging [8].

### Limitations

This study is limited by its single-center design, small study population, and voluntary participation. The nonresponse rate to the initial invitation was 89% (n=399), which may be due to mail delivery issues, lack of interest, or time constraints. While premalignant lesions were identified, no malignant lesions were imaged. Future studies that involve larger cohorts, different health care settings, and more teledermatologists could elicit additional information on the efficacy of direct-to-patient mobile teledermoscopy.

### Conclusions

Substantial agreement was found between direct-to-patient mobile teledermoscopy and in-office evaluation in the diagnoses and management of patient-identified lesions. Most participants reported ease with mobile teledermoscopy use; however, most lesions were benign, indicating the need for patient education on high-risk features to ensure appropriate lesions are imaged. Providing direct-to-patient mobile teledermoscopy services may expand the reach of existing teledermatology practice.

## Abbreviations

ABCDE: asymmetry, border, color, diameter, and evolving
SFVAMC: San Francisco Veterans Affairs Medical Center
